# Food consumption trends and drivers

**DOI:** 10.1098/rstb.2010.0149

**Published:** 2010-09-27

**Authors:** John Kearney

**Affiliations:** Department of Biological Sciences, Dublin Institute of Technology (DIT), Dublin, Eire

**Keywords:** food consumption trends, nutrition transition, globalization

## Abstract

A picture of food consumption (availability) trends and projections to 2050, both globally and for different regions of the world, along with the drivers largely responsible for these observed consumption trends are the subject of this review. Throughout the world, major shifts in dietary patterns are occurring, even in the consumption of basic staples towards more diversified diets. Accompanying these changes in food consumption at a global and regional level have been considerable health consequences. Populations in those countries undergoing rapid transition are experiencing nutritional transition. The diverse nature of this transition may be the result of differences in socio-demographic factors and other consumer characteristics. Among other factors including urbanization and food industry marketing, the policies of trade liberalization over the past two decades have implications for health by virtue of being a factor in facilitating the ‘nutrition transition’ that is associated with rising rates of obesity and chronic diseases such as cardiovascular disease and cancer. Future food policies must consider both agricultural and health sectors, thereby enabling the development of coherent and sustainable policies that will ultimately benefit agriculture, human health and the environment.

## 1. Introduction

Changes in agricultural practice over the past 50 years have increased the world's capacity to provide food for its people through increases in productivity, greater diversity of foods and less seasonal dependence. Food availability has also increased as a consequence of rising income levels and falling food prices. This has resulted in considerable changes in food consumption over the past 50 years. Along with an exploration of food consumption (availability) trends and projections to 2050, both globally and for different regions of the world, the drivers largely responsible for these observed consumption trends will be examined.

### (a) Sources of data for assessing food consumption trends

Several sources of data may be used when examining patterns in both the supply and consumption of foods for making worldwide comparisons or examining international trends over time. Such data may be derived from food balance sheets (FBSs), household budget surveys or individual dietary surveys (IDSs). Each of these methods has its own merits depending on the particular outcome desired. These methodologies are also discussed in another driver review (Hawkesworth *et al*. 2010). For the purposes of nutrition monitoring and surveillance, these same methodologies may also be employed with the exception of food production figures. Food production data may be useful when examining trends of specific food crops in different regions throughout the world. Production figures are available from FAO for every country in the world for every crop (Food & Agriculture Organization of the United Nations 2009). However, for the purposes of examining differences in food consumption patterns, it is not as accurate as data arising from FBSs.

#### (i) Food balance sheets

FBSs are constructed by the FAO from national accounts of the supply and use of foods, and are calculated from the food produced in and imported into countries minus the food exported net of imports, fed to animals or otherwise not available for human consumption, divided by the population size. FBS data provide information about average availability per person, that is they relate simply to the quantities of food reaching the consumer (i.e. a measure of availability). FBSs, while describing consumption of foods per capita of the population for a country, do not represent the amount of food actually consumed and will almost invariably result in an overestimation in food consumption compared with dietary surveys at the individual level (Serra-Majem *et al*. 2003). This point is also clearly illustrated in another driver review (Hawkesworth *et al*. 2010).

Annual FBSs tabulated regularly over a period of years (FAOSTAT database; see http://faostat.fao.org/site/354/default.aspx) will show the trends in the overall national food supply, disclose changes that may have taken place in the types of food consumed, i.e. the pattern of the diet, and reveal the extent to which the food supply of the country, as a whole, is adequate in relation to nutritional requirements. FBS data do not provide information on the variability within areas of a country or between different socio-demographic subgroups in the population. Those data are provided by individual cross-sectional dietary surveys at the national level. To determine the influences of socio-demographics, geography and the environment, it is necessary to examine the data from such national surveys. FBSs do not give any indication of the differences that may exist in the diet consumed by different population groups, e.g. different socio-economic groups, ecological zones and geographical areas within a country, nor do they provide information on seasonal variations in the total food supply. To obtain a complete picture, food consumption surveys showing the distribution of the national food supply at various times of the year among different groups of the population should be conducted. Nonetheless, only the FBS data can show long-term trends in food availability for a large number of countries as they are available for every country in the world, for every food item.

#### (ii) Individual dietary surveys

The monitoring of dietary patterns within countries is achieved by the use of nationwide monitoring systems. Such repeated cross-sectional studies—IDSs—are used to understand long-term changes in individual dietary intake. This information is used to identify trends in foods, nutrients and eating patterns among various subpopulations of interest. In the developed world, many countries conduct national surveys, and these provide an invaluable source of data for food and nutrient intake. Some countries, like the USA, conduct national surveys on a regular basis as a government requirement, but most do so less frequently owing to the large costs involved. In spite of this, national surveys are the major source of reliable information on actual dietary intake around the world. These are often supplemented by small surveys in single locations on smaller numbers of individuals.

Difficulties exist in making international comparisons in food intake as a result of variations in the methodology involved in ascertaining food intake. For example, in the USA, a 24 h recall method conducted by the US Department of Agriculture is used, while in the UK, a 7 day weighed food record is the method of choice. While assessment at the individual level through the use of IDSs provides the most accurate data on food actually consumed, they too have their limitations. Firstly, they are not available for all countries. Many countries, particularly in the developing world, do not have the resources (lacking the experience and expertise) to mount individual-level nutrition surveys. Such surveys are prohibitively expensive and labour-intensive. Secondly, and of particular relevance to this review, differences in methodology and data analysis greatly curtail one's ability to make meaningful comparisons of results at the international level. That is why food supply data have been presented in this review as they permit the examination of temporal trends and patterns at the broadest level geographically (and in particular between developing, industrialized and transition countries) as well as how these trends are likely to project towards 2050.

## 2. Global, regional and inter-country food consumption patterns

In addition to the data on commodity composition changes in available food consumption, in this review, data are also provided in terms of specific or individual food groups within the main food commodities, such as cereals, meat, livestock products (eggs and dairy foods), fish and vegetables, for different broadly defined regions of the world (electronic supplementary material, tables S1–S12). Furthermore, some within-country regional trends arising from IDS data are also presented in this review (tables 3–7). This, in part, serves to highlight the considerable inter- and intra-country variability and avoids the risk of oversimplification in the interpretation of the broader trends.

Globally, significant improvements have been made in raising food consumption per person with a rise of almost 400 kcal per person per day—going from 2411 to 2789 kcal per person per day between 1969/1971 and 1999/2001 (table 1) (Alexandratos 2006), and thus in the past four decades, dramatic improvement in reducing the prevalence of under-nutrition has taken place. There are still, however, some developing countries (especially in sub-Saharan Africa, e.g. Somalia, Burundi, Rwanda and Kenya) that have in fact declined further from what was already a very low per capita food consumption level. A detailed discussion on the levels of under-nourishment in countries throughout the world is provided in the FAO studies (Bruinsma 2003; Alexandratos 2006).

**Table 1.** Per capita food consumption (kcal per person per day). Reproduced with permission from Alexandratos (2006).

	1969/1971	1979/1981	1989/1991	1999/2001	2015	2030	2050
world	2411	2549	2704	2789	2950	3040	3130
developing countries	2111	2308	2520	2654	2860	2960	3070
sub-Saharan Africa	2100	2078	2106	2194	2420	2600	2830
Near East/North Africa	2382	2834	3011	2974	3080	3130	3190
Latin America and Carribean	2465	2698	2689	2836	2990	3120	3200
South Asia	2066	2084	2329	2392	2660	2790	2980
East Asia	2012	2317	2625	2872	3110	3190	3230
industrial countries	3046	3133	3292	3446	3480	3520	3540
transition countries	3323	3389	3280	2900	3030	3150	3270

In terms of calories arising from different major food commodities, large differences may be seen between the developing and industrial countries (table 2). While developing countries between 1963 and 2003 revealed large increases in the available consumption of calories from meat (119%), sugar (127%) and vegetable oils (199%), only vegetable oil consumption was seen to increase appreciably (105%) in industrial countries over these four decades. China, as a prime example of a populous developing country, showed even more dramatic changes in this 40 year period, especially in vegetable oils (680%), meat (349%) and sugar (305%) (table 2). In both developing and industrial countries (and again notably in China), declines were seen for pulses and roots and tubers between 1963 and 2003.

**Table 2.** Calories from major commodities (kcal per capita per day) in developing, industrial countries and China. Data from: FAOSTAT (http://faostat.fao.org/site/368/Desktop.Default.aspx?PageID=368#ancor).

		meat	% change four decades	sugar	% change four decades	pulses	% change four decades	roots and tubers	% change four decades	vegetable oils	% change four decades	wheat	% change four decades	rice	% change four decades
developing	1963	147		75		167		178		80		245		580	
countries	1983	210		128		113		157		145		453		694	
	2003	369	119	170	127	99	−41	154	−13	239	199	457	87	655	13
industrial	1963	833		349		40		145		241		592		188	
countries	1983	929		337		29		112		385		559		145	
	2003	958	15	328	−6	37	−7.5	112	−23	494	105	627	6	153	−19
China	1963	90		18		143		255		35		194		637	
	1983	192		54		50		222		95		534		962	
	2003	644	349	73	305	17	−88	176	−31	273	680	448	131	790	24

The marked rise in available food energy observed globally has been accompanied by changes in the composition of the diet. The process involved in such dietary change appears to follow a pattern involving two main stages. In the first stage, known as the ‘expansion’ effect, the main change is in terms of increased energy supplies, with these extra calories coming from cheaper foodstuffs of vegetable origin (Smil 2000). This development has been ubiquitous, occurring in both developed and developing countries. The second stage, called the ‘substitution’ effect, results in a shift in the consumption of foodstuffs with no major change in the overall energy supply. This shift is primarily from carbohydrate-rich staples (cereals, roots, tubers) to vegetable oils, animal products (meat and dairy foods) and sugar. In contrast to the first stage, this one is country-specific and is influenced by culture, beliefs and religious traditions. In particular, such traditions can influence the extent to which animal products substitute vegetable products and the specific types of meat and animal products consumed.

### (a) Cereals

Cereals continue to remain by far the most important food source in the world, contributing 50 per cent of calories and as much as 54 per cent in developing countries. Their contribution to energy intake varies markedly between developing and industrial countries. In developing countries such as in Africa and parts of Asia, cereals can contribute as much as 70 per cent of energy intake, while in industrial countries, for example, the UK, they provide approximately 30 per cent of energy intake and 50 per cent of available carbohydrates. Projecting to 2050, it is expected that the share of cereals in calories for food use will continue to decline slowly from 54 per cent in 2001 to 49 per cent in 2030 and 46 per cent in 2050 (Alexandratos 2006).

Trends and future projections of available food consumption for individual cereal categories are outlined in electronic supplementary material, tables S1 and S2. Globally, rice consumption (grams per capita per day) has seen negligible increases. This is due in large part to the declines in rice consumption in those countries that have predominantly rice-based diets (e.g. China and other East Asian countries). In those regions where consumption levels have historically been low (e.g. Oceania, North America, Africa and Europe), modest increases in consumption are projected, although these would still be far lower than the levels of intake in those regions with rice-based diets (e.g. 16 g per capita per day in Europe by 2050 versus 253 g per capita per day in Asia by 2050).

In contrast to this rather static situation for rice, global wheat consumption has increased at a faster rate than all other cereals. This growth is largely accounted for by the increase in developing countries (particularly in China, India) from the green revolution, reflecting increased crop yields. In terms of future projections, growth in wheat consumption will continue to be greatest in developing countries. This will be accompanied by continuing growth in wheat imports, especially in the non-producing countries or those countries favouring a dependence on diets that are made up of roots, tubers, bananas and plantains.

The consumption of coarse grains including millet and maize has been declining or remained largely unchanged in most regions of the world since the 1960s (electronic supplementary material, tables S1 and S2). Specifically, the available food consumption of millet has declined globally and particularly in Africa. Nonetheless, coarse grains (including sorghum) continue to be a major contributor to cereal consumption for many countries in sub-Saharan Africa. In world terms, maize consumption has increased and this increase is set to continue, albeit modestly, to 2050. Much of this increase is accounted for by the industrial countries, especially North America, which has seen developments in maize being used as sweeteners.

### (b) Meat

Meat has comprised an important part of the human diet for a large part of our history and still is the centrepiece of most meals in developed countries. In many developing countries, non-animal-based sources of protein are still dominant. In the USA and the UK, the most important meat sources are from pigs, sheep and cattle. In other regions such as India, the Middle East and those in Africa, goats and camels are the main meats consumed. In the UK, poultry (chicken) has now become the most popular meat source. Apart from the muscle, other parts of the animal collectively described as offal are also consumed. Meat products such as sausages, burgers, pork pies, etc., account for almost half of all meat consumed in developed countries.

There has been a considerable increase (62%) (electronic supplementary material, tables S3 and S4) in the available food consumption of meat worldwide, with the biggest increases in the developing countries (a threefold increase since 1963)—a considerable amount of this rise reflects the increases in Asia generally and China specifically. Unlike developing countries such as Brazil, which has seen a threefold increase, and China a dramatic ninefold increase in total meat consumption, it is not expected that countries such as India and Africa will see anything like these increases in the consumption of meat in the coming decades. The UK has one of the lowest intakes of red meat in Europe and consumption has been decreasing over the past 30 years. Contributors to this recent decline have been a number of food-related health scares, e.g. the bovine spongiform encephalopathy or ‘mad cow disease’ crisis. Globally, however, a considerable amount of the increase in meat consumption may be attributed to the increase in poultry consumption worldwide. Beef is the one meat group that on a worldwide level showed no increase in consumption levels during this time. This trend reflects the fact that while beef consumption rose modestly in some regions (in developing countries such as China and Brazil), it fell very modestly in most other regions (North America, Oceania and Europe). Projecting to 2050 suggests that the consumption of meat will increase moderately, and this will largely reflect increases in pork and particularly poultry.

### (c) Eggs, milk and other dairy products

Livestock products including eggs and dairy products such as milk, butter and cheese have shown variable consumption trends since 1963 (electronic supplementary material, tables S5 and S6). The levels of egg consumption (grams per capita per day) have doubled worldwide, with the increases more marked in developing countries compared with industrial countries. However, within these two categories of countries, considerable variability is apparent, with some developing countries such as India and many countries in sub-Saharan Africa showing little or no rise, and others such as Brazil and China experiencing quite marked increases in egg consumption. A similar picture of variability exists for the industrial countries, showing a modest rise in Europe, especially eastern Europe, a modest decline in North America and a sharper decline in Oceania (electronic supplementary material, tables S5 and S6).

While milk intake has risen in a number of developing countries, especially in Asia, in the USA, it has declined sharply over the past several decades, and this has been mirrored by a rise in the consumption of carbonated beverages and juices (Cavadini *et al*. 2000; Duffey & Popkin 2007). The future patterns of consumption to 2050 for these livestock products suggest that the consumption of eggs will continue to rise and the consumption of milk will continue to fall (at least in developed countries), while there will be no appreciable changes in butter and cheese consumption at the global level.

### (d) Fish

While fish catches worldwide are on the increase (Roberts *et al*. 2001), fish stocks are being depleted owing to over-fishing. The main fishes consumed are white fish, oily fish and seafood invertebrates. Fishes are an important source of good quality protein and are low in fat (except for the oily fish which provide a very good source of long-chain polyunsaturated fatty acids). Fishes may also be a major source of iodine accumulated from their environment. Compared with many European countries such as Portugal and Spain, consumption of fish in the UK is low at 22 g per capita per day (Office for National Statistics 2002).

Past trends in fish consumption for individual fish categories (categorized according to FAOSTAT) from 1963 to 2003 and future projections to 2050 are outlined in electronic supplementary material, tables S7 and S8. Globally, little or no increases were seen in the consumption (grams per capita per day) of demersal, marine or pelagic fishes. The main changes in consumption patterns may be seen for seafood and freshwater fishes, both of which have increased appreciably since the early 1960s. The highest increases in seafood have occurred in Oceania and Asia, especially China, with increases from approximately 11 g per capita per day in 1963 to approximately 69 g per capita per day in 2003. Compared with industrial countries, developing countries have also seen higher increases in freshwater fish consumption, with China having had the most marked increase in consumption with a 10-fold increase from 1963 to 2003 (electronic supplementary material, table S8). In terms of future trends, modest increases in pelagic fish consumption are predicted. The pelagic fishes are rich in long-chain omega-3 fatty acids of benefit to cardiovascular health. Many food-based dietary guidelines recommend increased intake of this particular food group.

Seafood consumption is set to continue to rise towards 2050 at a faster rate than any other fish category. Furthermore, intake of seafood will far exceed any of the other fish categories and such a trend is expected in both industrial and developing countries. Recommending an increase in fish consumption is one area where the feasibility of dietary recommendations needs to be balanced against concerns for sustainability of marine stocks.

### (e) Vegetables, roots, tubers, pulses and fruit

This group includes a wide range of plant families and consists of any edible portion of the plant, including roots (root crops), tubers, leaves, stems, buds, flowers and fruits. While fruit and vegetables do not make a significant contribution to macronutrient intake, they make an important contribution to dietary fibre. The legumes, especially the seed legumes, are of major nutritional importance, particularly in the developing world where in many countries they constitute a staple food along with cereals.

Consumption trends for roots and tubers (including cassava, sweet potatoes, potatoes, yams, taro and plantain) depict very modest declines worldwide and particularly in China and sub-Saharan Africa (electronic supplementary material, tables S9 and S10). Indeed, 19 countries within sub-Saharan Africa depend on these products for at least 20 per cent of their food consumption in terms of calories (Alexandratos 2006). This pattern reflects the sharp fall in the consumption of sweet potatoes in many developing countries accompanied by a parallel marked rise in the consumption of potatoes in a number of developing countries. This is especially apparent in China where consumption levels of sweet potato dropped from 227 g per capita per day in 1963 to 99 g per capita per day in 2003, while concurrently, the consumption of potatoes rose from 25 to 96 g per capita per day over the same time period. Contrasting patterns in the consumption of potatoes may be seen between industrial (falling levels) and developing countries (rising levels) (electronic supplementary material, tables S9 and S10). This highlights the fact that patterns observed for an overall food category (roots and tubers) are masking what is happening at the individual food level, i.e. the contrasting trends observed for sweet potatoes and potatoes. Thus, a much more complex picture is likely to exist than might be portrayed by observing trends in food category at its most aggregated level. Similarly, what is happening at the broad regional level does not give us the picture at country and obviously within-country level (IDS data are required for that). Pulses have declined in consumption levels globally and in particular among developing countries, e.g. a 10-fold drop in China from 30 g in 1963 to 3 g in 2003 (electronic supplementary material, table S10).

When discussing trends in fruit and vegetable consumption (electronic supplementary material, tables S9 and S10), it is important to remember that the data are referring to available rather than actual food consumption. Not to do so would be to describe an overly optimistic picture in terms of fruit and vegetable intake, with their combined intake far exceeding the recommended levels of at least 500 g or more per day. While production of fruits and vegetables has been increasing over recent years, inadequate consumption remains a problem worldwide. To increase consumption levels and address micronutrient deficiencies, apart from production increases by the horticultural sector, there needs to be a focus on adapting aspects of the market supply chain. This will help to make fruits and vegetables more accessible and affordable for poor households as well as ensuring access to markets by smaller producers.

### (f) Energy providers: vegetable oils, animal fats and sugar

The consumption patterns (historical trends and future projections) for these three energy providers in grams per capita per day are outlined in electronic supplementary material, tables S11 and S12. The consumption of vegetable oils has significantly increased in all regions of the world (threefold in developing countries and twofold in industrial countries). These increases in developing countries are most marked in China, Brazil and India (electronic supplementary material, table S12) and have therefore been significant contributors to increasing available food consumption (kcal per capita per day), thereby improving food security in these countries. It is envisaged that they are likely to increase still further among developing countries in the coming decades.

While animal fats were consumed at slightly higher levels than vegetable oils back in the early 1960s (most notably in Oceania and Europe), reverse trends have seen a marked decline in the consumption of animal fats in parallel with the rise in vegetable oil consumption. Such trends are likely to continue into the future, particularly in view of the health implications of diets high in animal fats, which tend to contain high proportions of saturated fatty acids that have implications for cardiovascular disease. Sugar, like vegetable oils, has seen marked increases in consumption among developing countries, most notably in Asia, India and to a lesser extent in Latin America and Africa (electronic supplementary material, table S12). However, inter-region and country differences exist with regard to trends, with some industrial regions such as North America and Oceania showing declines and others such as Europe (especially Eastern Europe and transition countries) showing modest gains.

### (g) Some inter-country trends in food consumption

In a study involving the comparison of FBS data and IDS data for four different countries: Canada, Finland, Poland and Spain, wide inter-country differences were seen for many food groups (Serra-Majem *et al*. 2003) (table 3). In comparison with the other countries in the study, more meat, potatoes and sugar were consumed in Poland, while in Finland, more dairy products were consumed (double that of Spain), and in Spain more fruits and vegetables, fish and pulses were consumed. Furthermore, this analysis highlighted the extent to which FBS data were seen to overestimate food consumption compared with IDS; for example, the consumption of dairy foods in Spain ranged from 430 to 226 g per person per day (a 43% overestimation). This level of overestimation, while similar between countries for dairy foods, was not apparent to the same degree for other food groups, or in the similarity in overestimation between countries.

**Table 3.** Mean consumption (grams per person per day) 1990/1992 of food groups derived from FBSs and IDSs in four countries. ND, no data. Reproduced with permission from Serra-Majem *et al*. (2003).

	Canada (North America)		Finland (Northern Europe)		Poland (Central Europe)		Spain (Southern Europe)	
	FBS	IDS	FBS	IDS	FBS	IDS	FBS	IDS
cereals	246	193	252	205	414	227	279	166
meats	273	141	180	134	210	236	278	173
dairy products	586	337	910	534	609	354	430	226
eggs	29	23	28	26	27	22	41	27
fish	61	37	89	43	32	ND	104	74
fruits	329	164	261	307	114	137	410	299
vegetables	296	146	152	92	307	288	417	211
roots and tubers	165	103	209	157	395	317	301	74
pulses	18	8	1	6	6	6	16	22
nuts and oil seeds	24	6	6	4	4	ND	33	4
oils and fats	69	33	50	39	68	57	82	30
sugar and honey	125	39	109	32	110	56	90	18

When inter-country comparisons are examined temporally, the picture becomes even more interesting (table 4). Research conducted for the Third Strategic Report of the Mediterranean Diet Surveillance System to examine 43 year time trends (1961/1965–2000/2004) in 41 countries using food availability data (FBSs) provided by the FAOSTAT database (Vareiro *et al*. 2009) found that European countries, especially those in the Mediterranean area, between the two time periods have undergone a ‘westernization’ of their food habits and have experienced a convergence in terms of non-Mediterranean food groups (table 4). Legumes, in contrast to many other food groups, have seen a marked decline, especially in Mediterranean Europe. All study regions saw an increase in vegetable oil, sugar and sweeteners as well as meat consumption over the past several decades. Northern Europe appears to be adopting a healthier dietary profile with increased fruit and vegetable consumption, fish and seafood as well as reductions in fat consumption (table 4). In southern, central and eastern European countries where fat intake was historically low, availability is currently rising.

**Table 4.** Mean availability of food groups (kcal per person per day) during the periods 1961/1965 and 2000/2004 in different regions of Europe. Reproduced with permission from Da Silva *et al*. (2009).

	Mediterranean Europe		Northern Europe		Central Europe	
	1961/1965	2000/2004	1961/1965	2000/2004	1961/1965	2000/2004
cereals	1279.1	1083.2	811.2	874.4	1278	1038.5
meats	148.5	354.4	280.7	420.7	257.7	356.7
animal fats	95	131.9	402.3	226.3	263.8	230.1
fish and seafood	24.9	44.8	41.1	21.3	12.5	19.1
fruits	120.1	135.2	78.7	104.2	99.4	99.5
vegetables	73.3	110	27.5	59.2	50.6	74.1
olive oil	115.4	127.1	1.5	13	2.29	7.58
pulses	72.9	49	14.6	20.3	24.6	18.4
nuts	28.3	34.1	8.4	16.5	15.9	21.6
vegetable oils	243.8	418.5	173.8	330.4	170.2	368.3
sugar and sweeteners	225.6	329.2	465.8	415.6	331.4	406.1

In southern, central and eastern European countries, fruit and vegetable consumption remains well below the recommended levels. This is also true in other developed and industrial regions such as Europe and Australia. For example, the WHO recommends that average fruit and vegetable intake should be at least 400 g of fruits and vegetables per person per day. But the dietary survey data show that adult intake of fruits and vegetables is less than this in 20 of the 25 countries for which data are available (table 5). Furthermore, in developed countries, a lower fruit and vegetable intake is observed among those of lower socio-economic status (SES) (Mullie *et al*. 2010). Such findings can only be picked up from IDS and not availability (FBS) data. Thus, having both sources of data is critical to giving us a more complete picture of food consumption patterns.

**Table 5.** Fruit and vegetable consumption in adults in selected European countries. ‘Vegetables’ does not include potatoes except in Italy and Germany. Sources: Anon. (1999); World Health Organization 2000, 2003).

	year of survey	age group surveyed	fruit (g per person per day)	vegetables (g per person per day)	fruit and vegetables (g per person per day)
Austria	early 1990s	19 and above	145	183	328
Azerbaijan	1994/1995	18 and above	121	46	166
Belgium	1980/1984	25–74	155	206	360
Croatia	1990	18 and above	157	142	299
Denmark	1995	19–64	115	159	273
Estonia	1997	18 and above	225	259	378
Finland	1992	25–64			433
France	1993/1994	19–64	202	187	288
Germany—West	1987/1988	18–88			244
Germany—East	1991/1992	18–80			349
Hungary	1992/1994	19 and above	201	159	360
Iceland	1990	18 and above	72	152	224
Ireland	1990	18 and above	118	111	229
Italy	1994/1996	18–60			433
Kazakhstan	1996	18 and above	130	35	168
Latvia	1997	19–64	183	83	266
Lithuania	1997	18 and above	189	170	359
Macedonia, Fmr Yug. Rep.	1996	18 and above	230	144	374
Norway	1993/1994	16–79	130	211	341
Portugal	1980	19–64	226	173	399
Slovenia	1997	18 and above	337	179	516
Spain—Catalonia	1992	18–60			480
Sweden	1989	15–74			265
Ukraine	1997	18 and above	190	87	285
UK	1986/1987	16–64			248
Uzbekistan	1984	18 and above	330	78	408

Da Silva *et al*. (2009) also found evidence of increasing westernization taking place with countries that traditionally had the highest adherence to the Mediterranean diet, most notably Greece, experiencing the greatest falls, while some countries in Northern Europe that originally had a very low adherence to the Mediterranean diet experienced a small increase in adherence (table 6). While globally our diets are becoming increasingly energy-dense and sweeter (with many higher fibre foods being replaced by processed versions), there is still huge heterogeneity in eating patterns that are region- and country-specific.

**Table 6.** Mean Mediterranean Adequacy Index for country groups and countries in 1961/1965 and 2000/2003. Reproduced in part with permission from da Silva *et al*. (2009).

	Mediterranean adequacy index	
country group (*n*)	1961–1965	2000–2003
world (169)	2.86	NA
Mediterranean countries (18)	3.44	1.28
Mediterranean Europe (10)	3.41	1.28
Central Europe (8)	1.71	0.82
Northern Europe (6)	0.83	0.67
country		
Greece	5.54	2.04
Egypt	4.81	4.09
Japan	4.11	1.51
Iran	2.87	3.65
Spain	3.35	1.19
Denmark	0.67	0.76
Germany	0.82	0.76
UK	0.68	0.87
USA	0.63	0.64
Australia	0.68	0.7
South Africa	1.87	1.78
Argentina	1.13	0.97

### (h) Trends in organic foods, functional foods, genetically modified foods

In describing historical and projected food consumption patterns in the context of overall food production, some mention of the trends in organic food production as well the consumption of functional foods including genetically modified (GM) foods is needed in order to provide a more comprehensive picture of food consumption trends.

#### (i) Organic foods

Organic food production places a strong emphasis on environmental protection and animal welfare. Recently, the demand for local, sustainable and organic food production has increased. Organic farming tends to improve biodiversity and sustainability within rural communities and has become one of the fastest growing segments of agriculture in many parts of the world with 82 per cent growth between 2006 and 2008 (Willer & Yussefi 2007). Currently, there are 30.4 million hectares worldwide certified according to organic standards, with Australia having the largest certified organic surface area of 12.3 million hectares, followed by China with 2.3 million hectares, Argentina with 2.2 million hectares and the USA with 1.6 million hectares (Paull 2008). On average, 5 per cent of EU land is being used for organic production, with the UK organic market the third largest after Italy and Germany (Willer & Yussefi 2007). Across Europe, there is a very high import rate (especially for fruits and vegetables) as the rate of production is far lower than the consumption demands for organic produce. This growing demand for organic products offers considerable opportunities for producers in developing countries (IFOAM 2008).

Consumer attitudes to organic foods are complex, often linking food to health, the environment, ethics and identity. Location of production plays a key role in promoting trust. A recent European survey on motives for purchasing organic foods found that ‘*it is healthier for them*’ (48%) and ‘*better for the environment*’ (16%) were the two most important reasons for selecting such foods (Walley *et al*. 2009). Consumers also believe that organic foods are more nutritious than conventional foods and are prepared to pay higher prices for them. Any nutritional differences or superiority of organic foods cannot be proven, however (Dangour *et al*. 2009). Indeed, on the basis of a very recent systematic review, no evidence of a difference in nutrient quality between organically and conventionally produced foodstuffs was found (Dangour *et al*. 2009). The higher price for organic foods (especially meat) can be attributed to reduced crop yields, higher cost of organic feed, lower animal stocking rates and higher labour requirements.

With the world's population rising and expected to reach 9 billion by 2050, a plan for the development of the organic sector is needed to meet this demand. However, the ability of organic agriculture to contribute significantly to the global food supply has been questioned owing to low yields, increased land use and insufficient quantities of organically acceptable fertilizers (Badgley & Perfecto 2007). It is unlikely therefore that organic agriculture would be capable of producing enough food to meet the expected increases in global food demand (Tilman *et al*. 2002). It is important therefore to find new solutions to the problems caused by growing populations and environmental degradation.

#### (ii) Functional foods

These may be defined as foods and food components that provide a health benefit beyond basic nutrition (quantities necessary for normal growth and development) and include conventional foods, fortified, enriched or enhanced foods and dietary supplements (Clydesdale 2004*a*,*b*).

Functional food consumption is increasing in almost all industrialized countries. Interest in functional foods and drinks has been fuelled by a desire for convenience, as well as health. Busier lifestyles are making it harder to meet nutritional requirements using traditional food and drinks. It is envisaged that the development of functional foods will continue to grow in industrialized countries, fuelled by increasing life expectancy, higher prevalence of non-communicable diseases, increasing healthcare costs and the acceptance of the strong link between diet and health. Nevertheless, consumers remain somewhat wary about health-related claims on food and drink products and sceptical of their efficacy. Success in the functional food market is increasingly dependant on establishing a relationship of trust with the consumer (Frewer *et al*. 2003).

#### (iii) Genetically modified foods

Proponents of GM foods see the benefits of recombinant DNA (r-DNA) technology as a tool offering potential benefits to farmers and consumers in a wide range of food and agriculture areas. They also see GM foods as offering the potential for a more abundant and economical food supply for the world. They point to the benefits through continued improvement in nutritional quality (including foods of unique composition for populations whose diets are lacking in essential nutrients), fresh fruits and vegetables with a longer shelf-life and the development of functional foods that may provide certain health benefits. On the other hand, those with particular concerns generally have fears about safety concerns regarding GM food, as well as environmental risks and ethical aspects of using r-DNA technology. The Cartagena Protocol on Biosafety (Secretariat of the Convention on Biological Diversity 2000) is an important step towards allaying environmental concerns in that it seeks to protect biological diversity from the potential risks posed by living modified organisms. It has also provided an important stimulus for the development of national genetically modified organisms (GMOs) regulatory frameworks in developing countries. This is important as much of the growth in the GM food market in the future will be in developing countries, most notably in Brazil and China.

In 2006, the countries that grew 97 per cent of the global transgenic crops were the USA (53%), Argentina (17%), Brazil (11%), Canada (6%), India (4%), China (3%), Paraguay (2%) and South Africa (1%). The majority of these crops were herbicide- and insect-resistant soya beans, corn, cotton, canola and alfalfa. In addition, for vegetable oil, representing one of the world's most important food commodities with a current annual production of 65 million tonnes, through r-DNA technology, the nutritive value (e.g. soya bean oil containing 80% oleic acid) and oxidative stability can be improved. Currently, GMOs are primarily an indirect food source, as the dominant crops in commercial use are used in livestock feed and food processing, and GM fish or livestock are not commercially available for food consumption.

#### (iv) The nutrition transition

Many of the changes in food consumption patterns discussed above are reflective of the nutrition transition—a series of adverse changes in diet, physical activity and health. The shift from a high prevalence of under-nutrition to a situation where nutrition-related non-communicable diseases (NR-NCDs) predominate arises where you have increased consumption of unhealthy foods along with increased prevalence of overweight and obesity in middle-to-low-income countries of the world. It can have serious implications in terms of public health outcomes, economic growth and international nutrition policy. The nutrition transition in a country is nearly always preceded by demographic and epidemiological transition.

China is experiencing the nutrition transition considerably sooner and also at a much lower level of gross national product (GNP) when compared with the USA and Western European countries (Popkin 1999). Furthermore, in China, the nutrition transition appears to be occurring at a faster rate among those on lower incomes. Now, owing to lower prices and an affluent food supply, even the poor have more access to (can afford) more fat and animal products. Another element of the nutrition transition is the increasing importation of foods from industrialized countries. As a result, traditional diets featuring grains and vegetables are giving way to meals high in fat and sugar. Rapidly transitioning countries (e.g. China, Mexico and Brazil), often referred to as emerging markets, are of particular concern. This is because these countries are experiencing very high rates of economic development, and the trend towards increased prevalence of overweight is now occurring most rapidly in conjunction with accelerated economic growth. Furthermore, it is expected that the nutrition transition will advance at a greater speed in these developing countries and that its consequences may be more severe and strongly felt (Popkin 2002).

**Figure 1.**
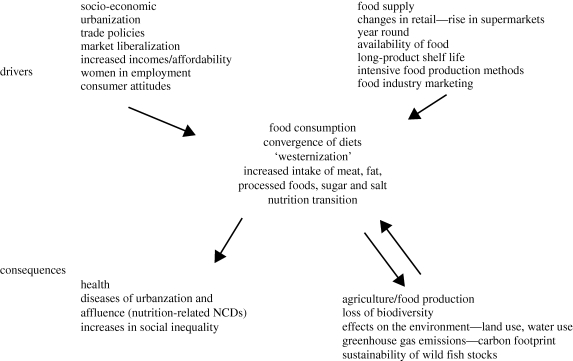
The drivers and consequences of food consumption changes with economic development.

A difficulty in arresting the effects of the nutrition transition is due in part to the paradox that while the diet associated with the nutrition transition (high fat, sugar and salt) is unhealthy, it is also more diverse and pleasurable (fat and sugar are two of the most pleasurable elements of the diet in terms of taste preferences). This then is part of the challenge: to provide more varied and tasteful diets while ensuring that these diets and a healthy activity level reduce the incidence in obesity, adult-onset diabetes and cancer related to nutrition and exercise. The relationship between recent food consumption patterns, some of their drivers and possible consequences are outlined in figure 1.

## 3. Drivers of food consumption

Food consumption is variably affected by a whole range of factors including food availability, food accessibility and food choice, which in turn may be influenced by geography, demography, disposable income, SES, urbanization, globalization, marketing, religion, culture and consumer attitudes. Some of these drivers that are specifically related to the nutrition transition are discussed below.

### (a) Income

Over the next three to four decades, global per capita income is projected to rise at a rate of over 2 per cent per annum, with developing countries that are starting from a low base expected to rise at even higher rates (Du *et al*. 2004). Their economies are expected to expand at twice the rate of those in industrial countries. Rising incomes means higher fat diets. In Mexico and Brazil, for example, where overweight used to be a sign of wealth, it now more often reflects poverty. Increased incomes or lower prices have lead to the increased consumption of animal-based foods and processed foods. While those that are well educated can choose to adopt a healthy lifestyle, the poor have fewer food choices and more limited access to nutritional education.

In China, when per capita income grew fourfold after the economic reforms of the late 1970s, the consumption of high-fat foods soared. In 1962, a diet containing 20 per cent of total energy from fat correlated with a per capita GNP of US$1475. By 1990, a GNP of just US$750 correlated with the same diet. Thus, while poverty has declined sharply in China between 1978 (20%) and 1998 (6%), the rise in income level has affected diets adversely from a health perspective. Data from a prospective Chinese dietary study (1989–1997) examined the impact of rapid income growth on food and nutrient intake (Du *et al*. 2004) (table 7). The effects of time and SES (income tertile) are apparent in the Chinese diet, moving from one high in carbohydrate foods to one high in fat and energy-dense foods (table 7). A substantive shift to a diet with more animal products (the average per capita intake of meat increased by 30 per cent between 1978 and 1997) and a lower consumption of traditional foods (rice, wheat and vegetables) has taken place. The consumption of edible oils increased sharply across all income tertiles, and this is partly explained by price, making them affordable even for low-income people. In addition, increased income has lead to increased income disparity and health inequality. Of concern from a health perspective is the fact that a projected doubling of income could lead to a 40 per cent increase in the incidence of high-fat diets in this population (Du *et al*. 2004).

**Table 7.** Consumption of some food groups according to income level in Chinese adults (20–45 years) 1989/1997. Reproduced with permission from Du *et al*. (2004).

	mean total consumption (g per capita per day)		proportion of consumers (%)		mean consumer intake (g per consumer per day)	
income tertile	1989	1997	1989	1997	1989	1997
flour and flour products						
low	177.4	218.5	52.3	72.4	339.4	301.7
medium	184.2	152	69.7	72.6	264.4	209.4
high	159.4	129.6	75.7	75.7	210.5	171.3
rice and rice products						
low	345.3	289.4	73.5	80.5	469.9	359.8
medium	315.5	306.7	82.2	90.7	383.8	338.2
high	299.8	273.1	90.9	93.6	329.7	291.7
animal source foods (pork, poultry, beef, mutton, fish, eggs, dairy)						
low	61.4	82.7	63.8	72.7	96.3	113.8
medium	107.2	137.5	82.8	88.8	129.5	154.9
high	145.1	209.1	91.9	97.1	157.8	215.2
edible oils (vegetable oils and animal fats)						
low	20.2	43.8	81.3	100	24.8	43.8
medium	24.4	43.9	85.5	100	28.5	43.9
high	25.2	51.6	85.1	100	29.6	51.6

In most industrial countries (e.g. the USA and the UK), the effects of increased income have generally been considered as beneficial, resulting in better quality diets, better healthcare, lower morbidity and mortality from infectious diseases and lower risk of obesity (Marmot 2001). The effects of SES on dietary intake have found disparities favouring a more healthy dietary pattern among those of higher SES in industrial countries over the past four decades (Lallukka *et al*. 2009). Part of this reflects a pattern of health-conscious behaviour resulting in health-driven behavioural change. It may also be that income is causally related to health through its effect on social participation and the opportunities to control life's circumstances (Marmot 2002).

### (b) Urbanization

Essentially, almost all of the population growth over the coming decades will be urban. In 1900, just 10 per cent of the world population inhabited cities. Today, that figure is over 50 per cent. While urbanization will proceed very slowly in many industrial and transition countries (such countries already being predominantly urban), it will continue to grow unabated in those countries where the vast majority of the country is rural. This is already particularly evident in sub-Saharan Africa (urbanization rate greater than 4%) and East Asia (urbanization rate greater than 3%). Urbanization has numerous consequences in that it leads to new and improved marketing (with greater access to modern mass media), distribution infrastructure, attracts large supermarkets dominated by multinational corporations, and results in better transportation systems thereby improving access to foreign suppliers and the importance of imports in the overall food supply (Hawkes 2006). This ultimately facilitates and results in the globalization of food consumption patterns.

Rapid urbanization has had, and will continue to have, a profound effect on food consumption patterns (Popkin 1999). A higher caloric intake (cities offer a greater range of food choices), combined with a lower-energy expenditure in urban jobs (with a reduction of physical activity of the order of 10–15%) compared with rural work and more inactivity in leisure time, means that obesity and diabetes in developing countries are advancing more rapidly in cities than in rural areas. Also, urbanization can affect food consumption by changes in dietary behaviour. This niche has been seized by the fast-food industry by providing quick access to cheap take-away meals. These meals also satisfy the consumers' demand for food high in salt, fat and sugar. Indeed, the most popular fast-food items, including hamburgers, pizzas and fried chicken, have 30 per cent of their food energy as fat (Smil 2000). Thus, the major consequences from a nutrition perspective of urbanization are a profound shift towards higher food energy, more fats and oils and more animal protein from meat and dairy foods. This results in a diet that is lower in fibre, vitamins and minerals and higher in energy, total and saturated fat. Data from the China Health and Nutrition Survey, which found an increase in the consumption of animal products, observed that this increase was higher for urban residents compared with those living in the countryside (Zhai *et al*. 2009). Furthermore, intake of animal foods was greater for urban (178.2 g per capita per day) compared with rural (116.7 g per capita per day) dwellers in 1997 (Zhai *et al*. 2009). Urbanization in the next few decades will primarily be a problem in developing countries (Mendez & Popkin 2004).

### (c) Trade liberalization

Trade liberalization is another important factor that has lead to changes in food intake. Modifications in food supply have also altered radically the food environment and the choices that consumers may make. Reductions in the price of unhealthy foods, typically those that are calorie-rich, nutrient-poor and high in saturated fats and salt, compared with healthy foods, increased desirability and availability of unhealthy foods, worsening asymmetry between consumers and suppliers of foodstuffs, and growing urbanization and changes in lifestyle are all possible means by which trade liberalization can affect food consumption, especially among poor populations (Thow 2009).

Trade liberalization can affect the availability of certain foods by removal of barriers to foreign investment in food distribution. It can also enable foreign investment in other types of food retail; multinational fast-food outlets have made substantial investments in middle-income countries. Availability of processed food has risen in developing countries after foreign direct investment by multinational food companies. Thus, changes in trade policies have facilitated the rising availability and consumption of meat, dairy products and processed foods (Thow & Hawkes 2009). These policies of trade liberalization therefore have implications for health by virtue of being a factor contributing to the ‘nutrition transition’ that is associated with rising rates of obesity and chronic diseases such as cardiovascular disease and cancer (Thow & Hawkes 2009).

While trade liberalization has enabled greater availability and affordability of highly processed, calorie-rich, nutrient-poor foods and animal products in developing countries, more research, however, is needed to better understand the relationship between trade policy and diets (Thow 2009). Although data are available on the growth of processed food sales (29% annual growth in developing countries versus 7% in nations with high incomes), evidence for consumption patterns of processed foods and their determinants in developing countries is still lacking (Hawkes 2005). Thus, more empirical data on the patterns of highly processed food consumption in developing countries are critically needed. It should also be borne in mind that it may be inadvisable to explore the trade and health relationship in isolation from a wider analysis of GNP growth and development.

### (d) Transnational food corporations *(*franchises and manufacturers*)*

Transnational food corporations (TFCs) (franchises and manufacturers) such as KFC, McDonalds, Kraft and Nestlé are all drivers of the fast-food market, processed foods and Western lifestyle that have become so widespread in developing countries (Hawkes 2005). Along with an increased consumption of modern processed foods from developed countries, developing countries are also creating processed versions of traditional dishes. Consequently, with the globalization of food systems, traditional diets in developing countries are being transformed as more meals are now available in the fast-food calorie-rich pattern of developed countries, and these are increasingly abundant and cheap through advances in food processing and modern technology.

Too often, however, sole blame is attributed to the globalization of modern food processing, marketing and distribution sectors including soft drink, fast-food and other multinational companies. Other factors that may also play a role include the rapid expansion of the global mass media, and changes that have brought about declines in energy expenditure related to leisure, work and transportation, as well as other factors related directly to the opening of our world economy (Popkin 2006).

### (e) Retailing

In a single globalizing decade from 1990 to 2000 and just into the liberalization of markets, changes have taken place in the retailing sector in Latin American that took North American retailing 50 years to accomplish (Reardon & Swinnen 2004). Supermarkets are now major players in most of the agri-food economy in Latin America. In 2000, supermarkets had approximately 60 per cent on average of the national retail sectors in South America and Mexico, a fourfold increase in a decade (Reardon *et al*. 2003). Indeed, supermarkets along with large-scale food manufacturers have profoundly transformed agri-food markets in this whole region. This rapid growth was only possible because supermarkets expanded beyond their original markets, moving into small and poor countries, from urban to rural areas.

This expansion of supermarkets now extends well beyond Latin America and is only about 5–7 years behind in East and South-East Asia as well as in Eastern and Central Europe (Reardon & Swinnen 2004). In those regions where supermarkets have made major inroads into the food retailing system, the entire food economy from farm to fork is affected. For the consumer, the consequences of supermarkets have seen many nutritional benefits with substantial improvements in the standards of food quality and safety (e.g. supermarkets solved the problem of keeping animal-based products chilled) at competitive prices. Cheap and most importantly safe milk became available to the poor in Brazil because of supermarkets. They also bring the advantage of convenience, a particularly attractive feature to the largely urban consumer. However, supermarkets may also lead to the increased availability of cheaper, less healthy food, being large providers of processed, higher fat, added-sugar and salt-laden foods, especially in developing countries.

### (f) Food industry marketing

Recent and radical changes in the food marketing and distribution system (through their globalization) have had a profound effect on food consumption patterns. The role of TFCs and the growth of supermarkets in developing countries, it has been argued, lie at the very centre of this development (Thow 2009). An example of how marketing as well as government subsidies can change patterns and trends of consumption can be seen from beverage consumption in the USA, for example, which has changed dramatically over the past 50 years. In 1945, Americans drank more than four times more milk than carbonated soft drinks; 50 years later, they were consuming nearly two and a half times more carbonated beverages than milk. The reasons for the increase in soft drink consumption have been advertising and heavy subsidies to the producers of corn syrup, which surpassed cane and beet sugar for the first time in 1985 (Putnam & Allshouse 1999). According to Willett (2002), exposure to TV advertising is perhaps the single largest factor responsible for the epidemic of obesity among children in the USA. Greater regulation of marketing and advertising of food, especially to children, is now receiving much more attention (Nestle 2002). Marketing has taken advantage of increased disposable incomes in countries such as China, India, Brazil and Mexico where consumers are spending more on foods that are often highly processed and unhealthy. This trend is compounded by the fact that many giant TFCs have launched aggressive marketing campaigns to penetrate consumer bases in these countries, precisely because of increased disposable income.

### (g) Consumer attitudes and behaviour

Consumer health awareness continues to grow with the increasing availability of health information going hand in hand with the ageing of populations and increased risk for lifestyle diseases. Selection of foods that are acceptable to an individual increasingly takes place in a context where availability is substantially influenced by the food industry and food retailers. If we want to be able to formulate policies that focus on reducing consumption of total energy or total fat, then we need to know why consumers eat more than they need to.

While a minimum consumption level is needed for survival, the levels of consumption seen by modern ‘Western’ culture vastly exceed those levels, ensuring that basic needs are met. Why? Juliet Schor in *The Overspent American* (Schor 1999) has made several insightful observations on the motivations for contemporary American consumers: ‘Consumer satisfaction depends less on what a person has in an absolute sense than on socially formed aspirations and expectations’. Anthropologist Willett Kempton (2001) has suggested that from an environmental perspective, a problem with consumption to display social status is that status is always relative, generating an unending spiral of increasing consumption, display and re-comparison.

While public interest in sustainability continues to rise and consumer attitudes are mainly positive, behavioural patterns are not always consistent with these attitudes. A study was recently undertaken to explore this consumer attitude–behaviour gap among Belgian consumers (Vermeir & Verbeke 2006). Involvement with sustainability, certainty and perceived consumer effectiveness was found to be directly associated with positive attitude and intention to buy sustainable food products (Vermeir & Verbeke 2006). Policies intent on improving healthier food consumption patterns need to pay attention to the role of consumers as drivers in food production as they have an important influence on the demand for various kinds of food products.

## 4. A brief discussion of the health impact of these food consumption trends

Having looked at the consumption patterns of foods and some of the drivers for the changing patterns observed, it is important to mention the potential implications to health in order to highlight the significant role that diet plays. This review does not explore the implications to the environment—not because they are insignificant (to the contrary), but rather because they are covered to a greater or lesser degree in other review papers in this volume.

The world is now increasingly being dominated by degenerative diseases. Indeed, there are now more overweight and obese people than underweight or malnourished in the world (Popkin 2006). It has also become apparent that NR-NCDs are emerging increasingly among lower- and middle-income groups in less affluent countries. Thus, in many of these developing countries, the spread of obesity has occurred in parallel with the globalization of food systems and the expansion of trade, foreign direct investment and TFCs.

In many upper- to middle-income developing economies (including countries such as Mexico, Brazil and South Africa), higher obesity prevalence is occurring among the lower SES groups and within populations that are also prone to under-nutrition. Thus, hunger among these lower SES groups is presenting itself through excessive consumption of energy-dense and nutrient-poor foods. The result then is a double burden of under-nutrition (from deficiencies of energy, micronutrients or both) and over-nutrition (energy-dense nutrient-poor diets leading to obesity and other NCDs). This double burden is also being observed at the household level as shown in a recent study in China (obesity levels there among adults are now in excess of 20%; Popkin 2009). Health policy in countries experiencing this double burden must therefore be careful to balance continued efforts at reducing under-nutrition with new policies targeted at reducing intake of highly processed, unhealthy foods. The recent changes in the composition of the diet towards a more energy-dense one rich in total and saturated fats (primarily from higher meat and milk consumption) are likely to lead to an increased prevalence of NCDs. Between 1961/1963 and 1999/2001, there has been a sharp rise in the number of countries exceeding the recommended levels for total fat (greater than 30%) and saturated fat (greater than 10%), such that over a third (62/178 countries in 1999/2001) were in excess of these recommendations (Popkin & Du 2003).

The consequent health burden arising from the nutrition transition is enormous. Increased consumption of highly calorific and more energy-dense food with less activity leads to an increased incidence of obesity and diet-related diseases like diabetes, coronary heart disease (CHD) and certain types of cancer (Shetty 2002). Popkin *et al*. (2001) conducted an analysis of diet trends in India and China and calculated the economic costs of these changes. While the incidence of under-nutrition by these estimates is declining, the incidence of obesity, diabetes and hypertension is rising. Countries such as India, Pakistan and Sri Lanka are experiencing epidemics in adult-onset diabetes (NIDDM) and CHD. Indeed, it is projected that by 2025, a fifth of diabetic patients will be Indian and three out of four will come from developing countries. In conclusion, a high intake of food (relative to energy expenditure), particularly energy-dense foods, will increase the likelihood of overweight and other non-communicable diseases and the associated costs to individuals and society. One recent study looking at the economic impact of obesity and diabetes in India and China found costs to be rising sharply to the extent that they represent a major component of GNP. Indeed, it has furthermore been suggested that it may even get to the point of actually overwhelming the health system of China (Popkin 2008).

## 5. Conclusion

Ageing, globalization and urbanization all represent new challenges to the achievement of a good nutrition status. The observed changes in dietary patterns brought about as a consequence of the rate and level of urbanization have significant effects on global food supply, markets and trade. This is particularly important in terms of the rise in over-nutrition (i.e. diet-related chronic disease) in many developing countries.

It is important when considering future food policy that a sustainable pattern of food consumption be considered, ensuring a sufficient supply of staples and of micronutrient-rich foods without encouraging excessive consumption of energy-dense, nutrient-poor foods. Food systems that diversify beyond subsistence farming and include fruits, vegetables, legumes and animal products result in improved nutritional status. ‘Healthy’ agriculture must be the goal whereby nutritional considerations become part of multinational agricultural policy-making, and at the same time, agricultural considerations must be incorporated into the improvement of nutrition and health. As Feenstra (2002) aptly put it—we should endeavour towards ‘A collaborative effort to build more locally-based, self-reliant food economies—one in which sustainable food production, processing, distribution, and consumption are integrated to enhance economic, environmental and social health’. Thus, food policies will only be effective if they are developed with input from both the agricultural and health sectors, thereby enabling the development of coherent policies that will ultimately be beneficial to agriculture, human health and the environment.
